# Evaluation of Different Initial Doses of Envarsus in De Novo Kidney Transplant Recipients

**DOI:** 10.3390/jcm14165687

**Published:** 2025-08-11

**Authors:** Patricio Más-Serrano, Antonio Franco, Iván Beltrá-Picó, Marcos Díaz-González, Claudia Colomer, Isabel Gascón, Elena de la Cruz, Javier Pérez-Contreras, Ricardo Nalda-Molina, Amelia Ramón-López

**Affiliations:** 1 Clinical Pharmacokinetics Unit, Pharmacy Department, Dr. Balmis University General Hospital of Alicante, 03010 Alicante, Spain; mas_pat@gva.es (P.M.-S.); beltra_iva@isabial.es (I.B.-P.); marcos.diaz.glez@gmail.com (M.D.-G.); colomer_cla@gva.es (C.C.); gascon_isa@gva.es (I.G.); 2 Institute of Health and Biomedical Research of Alicante, 03010 Alicante, Spain; perez_fra@gva.es (J.P.-C.); jnalda@umh.es (R.N.-M.); aramon@umh.es (A.R.-L.); 3 School of Pharmacy, Miguel Hernández University, 03550 San Juan de Alicante, Spain; 4 Nephrology Department, Dr. Balmis University General Hospital of Alicante, 03010 Alicante, Spain; ele.delacruz04@gmail.com

**Keywords:** tacrolimus, dose of Envarsus, kidney transplant, bioavailability

## Abstract

**Background/Objectives**: Envarsus is a novel prolonged-release formulation of tacrolimus with enhanced bioavailability. The summary of product characteristics recommends an initial dose of 0.17 mg/kg/day for the prophylaxis of rejection in kidney transplant recipients, which may be excessive. This study aimed to compare the pharmacokinetics of four different initial doses of Envarsus: 0.15 mg/kg/day (group 1), 0.12 mg/kg/day (group 2), 0.10 mg/kg/day (group 3), and 0.08 mg/kg/day (group 4). Induction therapy included thymoglobulin, sirolimus, and prednisone, with Envarsus initiated once serum creatinine levels fell below 3 mg/dL. **Methods**: A comprehensive pharmacokinetic sampling strategy was implemented between 48 and 72 h post-transplant, allowing for the calculation of AUC using the trapezoidal method. Additionally, trough levels at 72 h were assessed, with the therapeutic range defined as 5–8 ng/mL. Patients with trough concentrations above 8 ng/mL either had their tacrolimus dose reduced or their treatment temporarily discontinued for 24 h. Kidney function was evaluated three months post-transplant. **Results**: A total of 167 patients completed the study (39 in group 1, 43 in group 2, 42 in group 3, and 43 in group 4). The groups were balanced in baseline characteristics. Compared with groups 1 and 2, groups 3 and 4 had significantly lower mean trough concentrations (7.9 ng/mL and 6.5 ng/mL vs. 11.3 ng/mL and 10.8 ng/mL, respectively) and lower AUC values (310 ng·h/mL and 271 ng·h/mL vs. 458 ng·h/mL and 390 ng·h/mL, respectively). Additionally, the proportion of patients with supratherapeutic drug levels was lower in groups 3 and 4 (47.6% and 37.2% vs. 76.9% and 67.4%, respectively), as was the proportion of patients requiring a skipped dose (14.3% and 14.0% vs. 30.8% and 27.9%, respectively). Importantly, the percentage of patients within the therapeutic range was higher in the 0.08 mg/kg/day group (41.9%), demonstrating improved drug level stability at this dose. Despite these differences, kidney function remained similar in all groups at three months, and no significant differences in the incidence of adverse events were observed among the four dosing groups. **Conclusions**: An initial dose of 0.08 mg/kg/day resulted in adequate tacrolimus exposure, improved the proportion of patients within the therapeutic range, and minimized unnecessary drug accumulation. These findings suggest that a lower initial dose of Envarsus may be preferable to optimize drug exposure while improving therapeutic precision.

## 1. Introduction

Tacrolimus is currently a cornerstone of most immunosuppressive regimens in kidney transplantation, due to its well-documented efficacy in preventing allograft rejection [1]. However, patients on tacrolimus require close pharmacologic monitoring owing to the drug’s narrow therapeutic index and wide inter- and intra-individual pharmacokinetic variability. Because the area under the curve (AUC) and the trough concentration are strongly correlated, tacrolimus dosing can be individualized based on trough concentration alone, which simplifies the therapeutic drug monitoring process [2,3,4].

Tacrolimus is available in a twice-daily immediate-release formulation (Prograf, Astellas Pharma Inc, Tokyo, Japan), which has been proven effective in multiple studies [5]. However, because tacrolimus has poor water solubility, is metabolized in the intestinal tract, and P-glycoprotein in enterocytes limits absorption, bioavailability for Prograf in kidney transplant recipients is only around 17% [6]. In addition, poor adherence increases the risk of graft loss [7], and research indicates that patients are more likely to comply with once-daily regimens [8,9,10].

For these reasons, prolonged-release formulations of tacrolimus, which simplify treatment regimens and improve bioavailability, are likely to result in better post-transplant outcomes [11]. At present, there are two prolonged-release formulations of tacrolimus for once-daily administration: Advagraf (Astellas Pharma Inc., Tokyo, Japan), which has shown similar efficacy and safety to Prograf in several non-inferiority studies [12]; and the newer formulation, Envarsus, developed with MeltDose technology [13] to increase bioavailability.

Several studies have compared the pharmacokinetic profiles of tacrolimus through conversion of the three formulations in stable kidney transplant recipients [14,15,16]; or during the induction phase [11,17,18]. The results suggest that Envarsus is effective and safe. In the induction studies, Envarsus was administered at 0.17 mg/kg/day, as recommended in the summary of product characteristics. However, previous research in stable kidney transplant recipients suggests that this dose may be excessive, owing to the increased bioavailability of the drug [14]. This study compared four fixed starting doses of Envarsus to identify the lowest dose that achieves early target tacrolimus exposure, while describing renal function and safety outcomes over the first three months.

## 2. Methods

This study is a prospective, single-center, observational study conducted between 2020 and 2024. Participants were eligible if they were 18 years or older, Caucasian ethnicity, had no history of hepatitis C, received a kidney transplant from a deceased donor, were treated with Envarsus as part of induction therapy, and had a minimum follow-up period of three months. All eligible consecutive kidney transplant recipients fulfilling these criteria between January 2020 and December 2024 were included, ensuring an unselected study cohort.

The immunosuppressive regimen consisted of the following: thymoglobulin was administered at 1 mg/kg/day for the first three days, with subsequent adjustments based on peripheral blood T-cell count until renal function recovery, up to a maximum cumulative dose of 7 mg/kg for all recipients; sirolimus was administered at a initial dose of 2 mg/day to maintain blood levels between 4 and 5 ng/mL; prednisone was initiated at 1 mg/kg and tapered to 20 mg/day after one week and Envarsus was introduced once serum creatinine levels dropped below 3 mg/dL, and blood concentrations were guided by Model-Informed Precision Dosing to reach levels of 5–8 ng/mL.

Participants were stratified according to their initial Envarsus dose into four groups: 0.15 mg/kg/day (group 1), 0.12 mg/kg/day (group 2), 0.10 mg/kg/day (group 3), and 0.08 mg/kg/day (group 4). Initially, 43 transplant recipients were enrolled in group 1, receiving 0.15 mg/kg/day. After confirming that a lower dose was feasible based on the results of this group, an additional 43 patients were included in group 2, receiving 0.12 mg/kg/day. This stepwise approach continued, including 43 participants in groups 3 and 4.

Trough concentrations of Envarsus were measured at 72 h post-initiation, following three doses, and continued up to three months post-transplant. To quantify exposure, the trapezoidal area under the concentration–time curve (AUC_0–24h_) was calculated from a 10-point tacrolimus blood concentration curve starting at 48 h, corresponding to the third dose. Samples were collected at 0, 1, 2, 3, 4, 6, 8, 10, 12, and 24 h.

### Outcome Variables: The Analysis Considered Three Outcome Domains

*Pharmacokinetic end-points* comprised (i) C_trough_ 72 h, (ii) C_trough_/daily dose ratio, (iii) AUC_0–24h_, (iv) AUC_0–24h_/daily dose ratio, and (v) trough concentrations, which were categorized into three ranges: within the therapeutic range (5–8 ng/mL), above the therapeutic range (>8 ng/mL), and below the therapeutic range (<5 ng/mL). Patients with trough concentrations exceeding 10 ng/mL either skipped the next dose and resumed the following day at a reduced dose or had their subsequent tacrolimus dose adjusted immediately. Those with concentrations between 8 and 10 ng/mL had their dose reduced accordingly.

*Efficacy* was evaluated as renal-function evolution evaluated three months post-transplant using serum creatinine levels and the estimated glomerular filtration rate (GFR), calculated with the Chronic Kidney Disease Epidemiology Collaboration (CKD-EPI) equation.

*Safety end-points*, recorded throughout the three-month follow-up, included nephrotoxicity (defined as acute CNI-related kidney dysfunction, i.e., a rise in serum creatinine ≥0.3 mg dL^−1^ within 48 h or ≥25% above baseline within 7 days after tacrolimus initiation, in the absence of rejection, obstruction, or volume depletion, and with partial reversal after tacrolimus dose reduction), infectious complications (cytomegalovirus or BK infection and admission for bacterial infection), and clinical manifestations of tacrolimus toxicity (neurotoxicity or tacrolimus withdrawal due to toxicity). A graft biopsy was performed whenever graft function deteriorated without an obvious cause.

Sex distribution, body weight, age, cold ischemia time, and immunologic risk, assessed by the percentage of pretransplant panel-reactive antibodies (PRAs), were evaluated across the four study groups. The incidence of delayed graft function (defined as the need for at least one dialysis session within the first 7 days after transplantation, excluding cases of polyuric acute tubular necrosis that did not require dialysis) was also recorded (Table 1).

Whole-blood Envarsus concentrations were measured using an enzyme immunoassay, using affinity chrome-mediated immunoassay (ACMIA) on the Tacrolimus Siemens platform (Dimension EXL 200). The measurement range was 1.0–30.0 ng/mL, with a limit of quantification (LoQ) of 1.0 ng/mL and a limit of detection (LoD) of 0.7 ng/mL.

Continuous variables were reported as means with 95% confidence intervals (CIs) or medians with inter-quartile ranges (IQRs), according to their distribution, assessed by the Shapiro–Wilk test. Group comparisons used one-way ANOVA for normally distributed data or the Kruskal–Wallis test for non-normal data; when the global test was significant, pair-wise contrasts were evaluated and *p*-values were adjusted with the Bonferroni method (α_adj_ = 0.05/number of comparisons), replacing the originally planned Tukey HSD. Categorical variables were expressed as percentages and compared with the χ^2^ test (or Fisher’s exact test when appropriate); significant χ^2^ results were followed by Bonferroni-corrected pair-wise analyses based on adjusted standardized residuals. Statistical significance was set at α = 0.05 (two-sided). All analyses were performed with SPSS version 24.

This study complied with the principles of the Declaration of Helsinki and was approved by the Ethics Committee of Dr. Balmis General University Hospital.

## 3. Results

A total of 172 patients were included, of whom 64.7% were male and 35.2% were female. The median (IQR) age of the population was 64.0 years (53.0–71.0) with a body weight of 70.0 kg (60.5–80.0). Two patients experienced early graft loss unrelated to biopsy-proven rejection, and three were lost to follow-up. Therefore, 167 patients completed the study, with 39 in group 1 (0.15 mg/kg/day), 43 in group 2 (0.12 mg/kg/day), 42 in group 3 (0.10 mg/kg/day), and 43 in group 4 (0.08 mg/kg/day) (Table 1).

The four groups were comparable in sex distribution, body weight, age, cold ischemia time, and immunological risk, with no statistically significant differences among them (Table 1). The proportion of male participants varied between groups, ranging from 48.7% in group 1 to 76.2% in group 3, but this difference was not significant. The median (IQR) weight was slightly higher in groups 3 and 4, with values of 72.5 kg (65.0–79.5) and 73.0 kg (65.0–80.0), respectively, compared to groups 1 and 2, with weights of 66.0 kg (58.0–80.0) and 68.0 kg (57.0–82.0). The median age was lower in group 4 (59.0 years (50.0–65.0)) than in the other groups (median range: 62–67 years) (*p* = 0.016).

**Figure 1:**
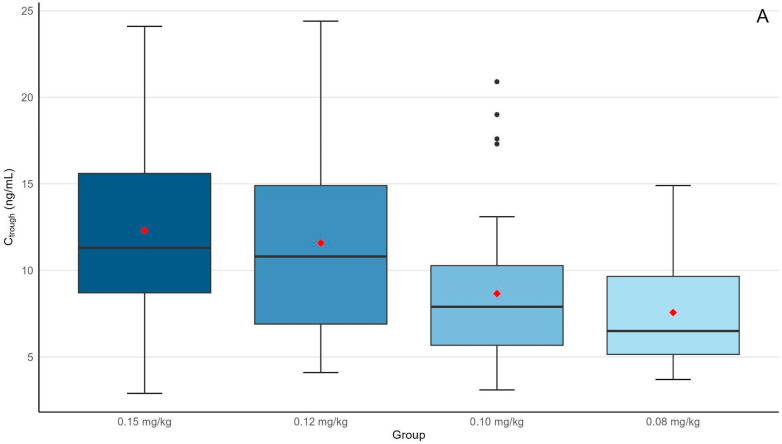

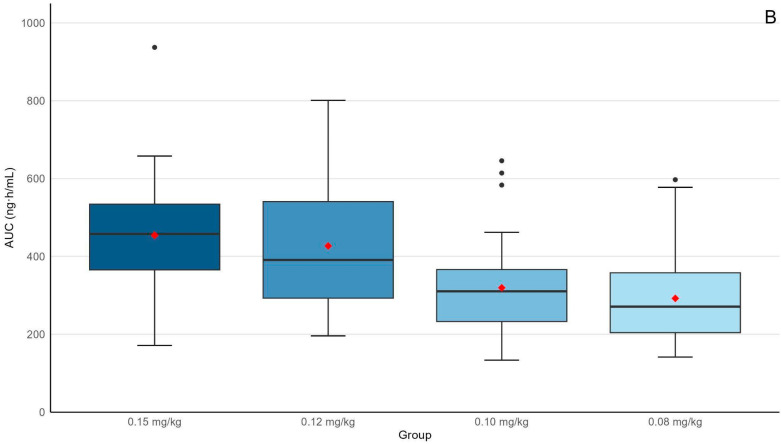
Boxplot of trough concentrations (C_trough) (**A**) and area under the concentration–time curve (AUC_0–24h_) (**B**) measured at 72 h post-administration in the four dosing regimens (0.15 mg/kg/day, 0.12 mg/kg/day, 0.10 mg/kg/day, and 0.08 mg/kg/day). The red diamond indicates the mean value for each group.

Table 2 summarizes the pharmacokinetic parameters and efficacy outcome (median, (IQR)). The initial dose of Envarsus was significantly different between groups, with the highest dose in group 1 (10.0 mg (9.0–12.0)) and the lowest in group 4 (6.0 (5.0–6.5)) (*p* < 0.001). The corresponding weight-based doses were 0.15 mg/kg (0.14–0.15) in group 1, 0.12 mg/kg (0.12–0.12) in group 2, 0.10 mg/kg (0.10–0.10) in group 3, and 0.08 mg/kg (0.08–0.08) in group 4. Trough concentrations at 72 h showed a progressive decrease with dose reduction, from 11.30 ng/mL (8.70–15.60) in group 1 to 6.50 ng/mL (5.15–9.65) in group 4 (Bonferroni-adjusted pair-wise comparison showed significant differences between groups 1 and 3 (*p* = 0.0030), groups 1 and 4 (*p* < 0.001), and groups 2 and 4 (*p* = 0.0012)). Similarly, AUC values decreased from 458 ng·h/mL (366–534) in group 1 to 271 ng·h/mL (204–358) in group 4 (Bonferroni-adjusted pair-wise comparison showed significant differences between groups 1 and 3 (*p* < 0.001), groups 1 and 4 (*p* < 0.001), and groups 2 and 4 (*p* < 0.001)) (Figure 1). Both trough concentration and AUC were significantly lower in groups 3 and 4 compared with groups 1 and 2. However, there were no significant differences between groups 3 and 4 or between groups 1 and 2.

The trough concentration-to-dose ratio (C/D ratio) and AUC normalized to dose (AUC_0–24h_/D ratio) did not differ significantly among the groups (1.14, 1.27, 1.07, and 1.11 ng/mL/mg, and 42.4, 48.9, 43.0 and 47.2 ng·h/mL/mg, for groups 1 through 4, respectively). Renal function at three months post-transplant was similar in all groups. The median (IQR) serum creatinine values ranged from 1.38 mg/dL (1.14–1.78) in group 1 to 1.48 mg/dL (1.25–1.77) in group 3, with no significant differences. Likewise, glomerular filtration rate (GFR), estimated using the CKD-EPI equation, showed no significant differences, with rates of 48.6 mL/min (35.2–59.1) in group 1, 43.2 mL/min (35.1–50.7) in group 2, 44.0 mL/min (36.4–51.9) in group 3, and 44.4 mL/min (38.9–50.7) in group 4. No biopsy-proven acute rejection episodes occurred in any of the study groups during the follow-up period.

**Figure 2:**
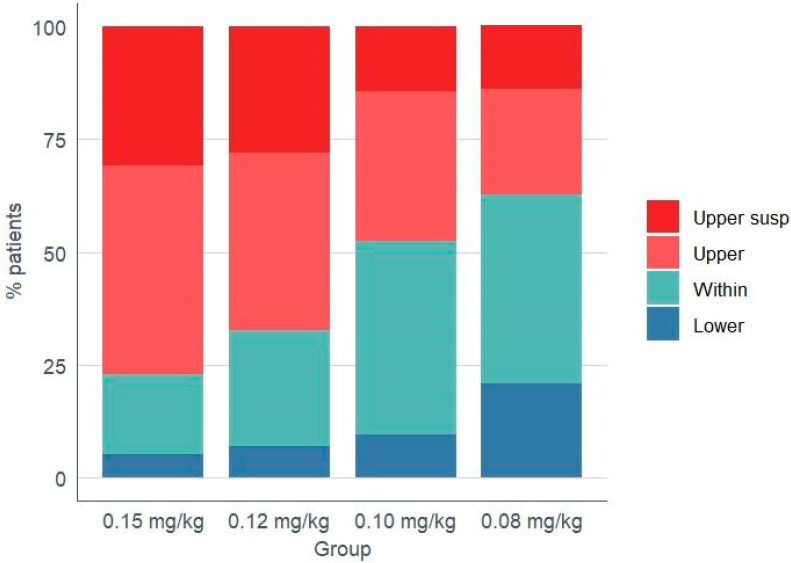
Stacked column chart. The bars represent the distribution of the percentage of the total number of cases in each category (lower, inside, upper, or upper skipped) for the four groups studied.

Table 3 shows the percentage of patients in each group according to their tacrolimus trough concentrations at 72 h, categorized as below, within, and above the therapeutic range. A global Fisher–Freeman–Halton test revealed a clear inter-group difference for supratherapeutic levels (*p* = 0.001) and a weaker, borderline signal for concentrations within the therapeutic window (*p* = 0.037), whereas subtherapeutic levels were comparable across cohorts (*p* = 0.085). After Bonferroni adjustment for six pair-wise comparisons (α_adj_ = 0.008), only the contrast between the highest and lowest starting doses remained statistically significant, with 76.9% of patients in group 1 (0.15 mg/kg/day) versus 37.2% in group 4 (0.08 mg/kg/day) exhibiting supratherapeutic concentrations (p_adj_ = 0.002). The same category showed near-significant differences for group 1 versus group 3 (p_adj_ = 0.068) and group 2 versus group 4 (p_adj_ = 0.055), while within-range exposure differed nominally between groups 1 and 3 (raw *p* = 0.018) but lost significance after correction (p_adj_ = 0.107). Consistently with the pharmacokinetic pattern, tacrolimus doses were skipped because of high trough levels in 30.8% and 27.9% of patients in groups 1 and 2, respectively, compared with 14.3% and 14.0% in groups 3 and 4, although this overall difference did not reach statistical significance (*p* = 0.124). Figure 2 shows the distribution of concentration categories and accompanying dose-skipping events across the four dosing regimens. Dose skipping was decided exclusively on pharmacokinetic results; no patient required interruption because of clinical signs of tacrolimus toxicity, and in every case a single dose was sufficient to restore target concentrations. The cumulative incidence of adverse events—nephrotoxicity, neurotoxicity, cytomegalovirus (CMV) infection, BK virus infection, admission for bacterial infection, and tacrolimus withdrawal—did not differ significantly between groups (Table 4). Finally, there were more patients within the therapeutic range in groups 3 and 4 (42.9 and 41.9, respectively) compared to groups 1 and 2 (17.9 and 25.6, respectively). The proportion of patients with subtherapeutic trough concentrations was highest in group 4 (20.9%); however, dose adjustments in these cases were minor, with no patient requiring an increase of more than 30% to reach the target therapeutic range.

## 4. Discussion

Recently, Veloxis Pharmaceuticals introduced Envarsus, a novel tacrolimus formulation developed using MeltDose technology, which enhances the release and absorption of drugs with poor water solubility [13]. This improved bioavailability suggests that the required induction dose of Envarsus may be lower than that of other once-daily tacrolimus formulations.

Although the initial dose recommended in the summary of product characteristics and tested in multiple induction studies is 0.17 mg/kg/day [11,17,18], the transplant protocol at our center employs a lower dose based on findings from a conversion study conducted by our team in stable kidney transplant recipients who had previously received immediate- and prolonged-release tacrolimus formulations [14]. Moreover, thymoglobulin is routinely administered as part of induction therapy in our hospital, minimizing the risk of inadequate immunosuppression in patients who do not initially reach therapeutic tacrolimus levels, and thymoglobulin is discontinued only when patients achieve therapeutic tacrolimus levels, independently of the initial dose. This strategy explains why renal function outcomes did not differ between the four study groups, as no recipient was exposed to insufficient immunosuppression.

The four study groups were comparable in terms of age, sex, body weight, cold ischemia time, donor age, PRA, HLA mismatches, and delayed graft function (Table 1). Since body weight was similar in the four groups, it is unlikely to have influenced the bioavailability of Envarsus. It has been reported that body mass index can affect the absorption and metabolism of tacrolimus, so a significant difference in weight could have impacted the results [19]. The secondary immunosuppressive agents, sirolimus and prednisone, were administered at similar doses in all groups. Additionally, none of the participants had hepatitis C or used medications that could interfere with Envarsus metabolism. The doses administered to all patients were scrupulously adjusted to the study design (Table 1).

The therapeutic range for Envarsus at our protocol was determined considering two key factors: the inclusion of thymoglobulin in all patient induction regimens and the higher immunosuppressive potency of mTOR inhibitors compared with mycophenolate, as demonstrated in the TRANSFORM [20] and ADHERE [21] trials. These studies defined lower target tacrolimus levels for patients treated with mTOR inhibitors, similar to those in our population, compared to those receiving mycophenolate mofetil.

The categorical analysis demonstrated a statistically significant high percentage of patient with supratherapeutic exposures in group 1 (0.15 mg/day, 76.9%) than in group 4 (0.08 mg/kg/day, 37.2%; p_adj_ = 0.002). Moreover, the proportion of patients who achieved concentrations within the therapeutic window was numerically higher in group 4 than in group 1 (41.9% vs. 17.9%), although with no statistical differences (p_adj_ = 0.107) (Table 3). Median C_trough_ and AUC0–24 did not differ significantly between the two higher-dose groups (0.15 vs. 0.12 mg/kg/day) or between the two lower-dose groups (0.10 vs. 0.08 mg/kg/day). These results probably reflects two real-world features of our cohort: first, in our study, we down-titrated the starting dose more aggressively in groups 3 and 4 (≈33% reduction) than in groups 1 and 2 (≈20%), narrowing inter-group pharmacokinetic separation; second, there was a potential type 2 error due to the limited sample size. These observations highlight the narrow therapeutic window of tacrolimus and reinforce the importance of initiating therapy with the lowest possible dose that still achieves therapeutic levels. A lower starting dose minimizes the risk of early overexposure and reduces toxicity, while also offering economic benefits [22].

The absence of between-group differences in adverse events is consistent with the fact that protocol-driven dose divergence lasted ≤72 h and that subsequent Model-Informed Precision Dosing enabled individualized dose titration to maintain trough concentrations within the target range, resulting in comparable cumulative exposure across all cohorts.

From a pharmacokinetic standpoint, the most accurate measure of tacrolimus exposure is AUC, as it reflects the total blood concentration of the drug over time [23]. However, few studies have evaluated Envarsus exposure in kidney transplant recipients immediately post-transplant using 24 h AUC as the primary parameter, particularly with calculations based on the trapezoidal method. Most previous studies have focused on stable transplant recipients. Tremblay et al. [24] conducted a conversion study of immediate-release tacrolimus to prolonged-release formulations in stable patients, reporting an AUC_0–24h_ of 262.0 ng·h/mL with a median dose of 4.8 mg/day. Similarly, Woillard et al. [25] reported AUC_0–24h_ ranging from 192 to 288 ng·h/mL in kidney transplant recipients, calculated using Bayesian estimation, with a median dose of 5 mg/day of different formulations during the first year post-transplant. Baraldo et al. [13] reported AUC_0–24h_ of 206.7–212.1 ng·h/mL in kidney transplant recipients and 185.4–196.41 ng·h/mL in liver transplant recipients receiving Envarsus. In other studies, AUC_0–24h_ values have been normalized to the AUC_0–24h_/D ratio. In the ASERTAA study [26], patients receiving daily doses of 0.103–0.121 mg/kg had an AUC_0–24h_/D ratio of 36.37 ng·h/mL/mg two weeks post-transplant. Similarly, Kamar et al. [11] reported an AUC0–24/dose ratio of 45.6 ng·h/mL/mg on day 3 with an initial dose of 0.16 mg/kg/day; our 0.15 mg/kg/day cohort showed a virtually identical ratio (45.9 ng·h/mL/mg) but a lower total AUC (≈ 454 vs. 552 ng·h/mL), consistent with the slightly smaller dose and lower trough concentrations in our population.

The AUC_0–24h_ values in our study for the lowest dose (0.08 mg/kg/day) align with the literature-recommended target of 300 ng·h/mL for kidney transplant recipients [23]. These results underscore the importance of AUC as a key metric for personalizing tacrolimus dosing to minimize overexposure and adverse effects while optimizing clinical outcomes.

Some studies have included a significant proportion of Black transplant recipients [4,27,28,29], who may require up to twice the dose of tacrolimus compared to White recipients to achieve similar trough concentrations [30]. Tremblay [15] highlighted this racial difference in tacrolimus pharmacokinetics. Our study population, similar to that described by Kamar [11], consisted exclusively of White patients from a geographically limited area, minimizing potential confounding due to genetic variability.

Although previous reports have demonstrated that the expression of CYP3A5*1 in either donors or recipients can significantly increase tacrolimus clearance, our decision not to genotype was supported by data from a similar cohort of 425 patients [31]. In that study, only 2.4% of patients were homozygous for CYP3A5*1, and 16.9% were heterozygous, while the vast majority (80.7%) were homozygous for CYP3A5*3. This distribution, consistent with a predominantly Caucasian population, suggests that the influence of CYP3A5*1 on tacrolimus metabolism is minimal in our cohort. Given the low frequency of CYP3A5*1 carriers and the consequently limited impact on tacrolimus pharmacokinetics, we did not include pharmacogenetic testing in our study. As observed in other studies with homogeneous populations, the modest number of rapid metabolizers and our limited sample size would not have provided sufficient statistical power to draw robust conclusions regarding tacrolimus clearance.

Our study has some limitations. As a single-center prospective observational study, the findings may not be generalizable to a broader population. Additionally, the groups were not parallel, although they were well balanced and recruitment occurred within close temporal proximity. However, selection bias could not be precluded due to the design of the study. Another limitation is the lack of genotyping to identify the proportion of slow and rapid metabolizers in each group; however, the close agreement in C/D ratio and AUC_0–24h_/D ratio between groups suggest comparable tacrolimus clearance. Finally, some numerically important contrasts (e.g., the proportion of patients within the therapeutic window) did not reach statistical significance once Bonferroni correction was applied, so larger multicenter prospective studies are needed to rule out a potential type 2 error. A key strength of our study, compared to multicenter observational studies, is the precise individual dosing data available for all patients, including exact administration times and blood sampling, per our standardized transplant protocol. Furthermore, because all transplants were performed at the same hospital, surgical techniques, immunological assessments, and nephrology protocols remained consistent across all participants. This consistency was further strengthened by the integration of Model-Informed Precision Dosing in individualizing dosing, which played a crucial role in optimizing tacrolimus therapy, ensuring individualized immunosuppressive management and reducing variability in patient outcomes.

## 5. Conclusions

The results of the present study, conducted under a center-specific immunosuppressive protocol, show that the lowest fixed starting dose evaluated (0.08 mg kg/day) yielded the highest proportion of patients within the therapeutic window while limiting early supratherapeutic exposure. Renal function and the cumulative incidence of adverse events (nephrotoxicity, neurotoxicity, CMV infection, BK virus infection, admission for bacterial infection, and tacrolimus withdrawal) at three months were comparable across all dose groups, highlighting the importance of early Model-Informed Precision Dosing and individualized titration. These findings support a “start-low, go-slow” strategy with Envarsus and warrant the prospective evaluation of even lower initial doses in larger cohorts.

## Figures and Tables

**Table 1:** Demographic characteristics.

Variable	Group 1 (n = 39) [0.15 mg/kg]	Group 2 (n = 43) [0.12 mg/kg]	Group 3 (n = 42) [0.10 mg/kg]	Group 4 (n = 43) [0.08 mg/kg]	*p*
Sex (men) (n (%))	19 (48.7)	29 (67.4)	32 (76.2)	28 (65.1)	0.074
Body weight (kg)	66.0 (58.0–80.0)	68.0 (57.0–82.0)	72.5 (65.0–79.5)	73.0 (65.0–80.0)	0.302
Age (yrs)	62.0 (54.0–74.0)	67.0 (58.0–74.0)	66.0 (58.0–71.0)	59.0 (50.0–65.0)	0.016
Donor age (yrs)	53.2 (50.4–56.1)	54.6 (51.6–57.1)	52.6 (50.1–53.8)	53.7 (50.7–56.8)	0.687
Cold ischemia (h)	15.8 (12.4–18.3)	15.4 (11.7–17.3)	16.4 (12.4–17.8)	16.1 (11.9–19.7)	0.636
PRAs > 50 (n (%))	3 (7.7)	4 (9.3)	3 (7.1)	3 (7.0)	0.980
HLA mismatches (n (%))	2 (5.1)	3 (7.0)	2 (4.8)	2 (4.7)	0.960
Delayed graft function (n (%))	5 (12.8)	5 (11.8)	8 (19.0)	5 (11.6)	0.720
Delayed tacrolimus start (n (%))	23 (59.0)	19 (44.2)	24 (57.1)	18 (41.9)	0.281
Delayed tacrolimus start (days)	4 (3–6)	3 (2–9)	4 (2–11)	3 (2–7)	0.431
Dose (mg)	10.0 (9.0–12.0)	8.0 (6.8–10.0)	7.00 (6.0–8.0)	6.0 (5.0–6.5)	<0.001
Dose (mg/kg)	0.15 (0.14–0.15)	0.12 (0.12–0.12)	0.09 (0.10–0.10)	0.08 (0.08–0.08)	<0.001

Median (IQR 25–75); PRAs, percentage of panel-reactive antibodies before the transplant. The Fisher–Freeman–Halton exact test was used for the categorical variables (two-sided), and for the continuous variables, the Kruskal–Wallis test was employed.

**Table 2:** Pharmacokinetic and efficacy outcomes.

Variable	Group 1 (n = 39) [0.15 mg/kg]	Group 2 (n = 43) [0.12 mg/kg]	Group 3 (n = 42) [0.10 mg/kg]	Group 4 (n = 43) [0.08 mg/kg]	*p*
Ctrough (ng/mL)	11.3 (8.7–15.6)	10.8 (6.9–14.9)	7.9 (5.6–10.3) *	6.5 (5.2–9.7) **^,‡^	<0.001
C/D ratio (ng/mL/mg)	1.14 (0.85–1.45)	1.27 (1.06–1.74)	1.07 (0.88–1.49)	1.11 (0.95–1.60)	0.277
AUC_0–24h_ (ng × h/mL)	458 (366–534)	3901 (293–541)	310 (223–366) *	271 (204–358) **^,‡^	<0.001
AUC_0–24h_/D ratio (ng × h/mL/mg)	42.4 (36.0–55.2)	48.9 (40.0–63.8)	43.0 (34.0–57.3)	47.2 (35.5–58.6)	0.169
Cr (mg/dL, 3 m)	1.38 (1.14–1.78)	1.67 (1.40–2.24)	1.72 (1.20–2.15)	1.48 (1.25–1.77)	0.115
GFR CKD-EPI (ml/min, 3 m)	48.6 (35.2–59.1)	43.2 (35.1–50.7)	44.0 (36.4–51.9)	44.4 (38.9–50.7)	0.329

Median (IQR 25–75); Ctrough, blood concentration of tacrolimus post 72 h; C/D ratio, blood concentration of tacrolimus post 72 h/daily dose; AUC_0–24h_, area under concentration; AUC_0–24h_/D ratio, area under concentration/daily dose; Bonferroni-adjusted pair-wise comparisons (αadj = 0.008, six contrasts per category). Only adjusted *p*-values are shown. Ctrough: significant differences were found between * groups 1 and 3 (*p* = 0.0030), ** groups 1 and 4 (*p* < 0.001), and ^‡^ groups 2 and 4 (*p* = 0.0012). AUC_0–24h_: significant differences were found between * groups 1 and 3 (*p* < 0.001), ** groups 1 and 4 (*p* < 0.001), and ^‡^ groups 2 and 4 (*p* < 0.001).

**Table 3:** Patients with tacrolimus trough concentrations at 72 h being lower, within, or higher than the therapeutic range.

	Group 1 (n = 39) [0.15 mg/kg]	Group 2 (n = 43) [0.12 mg/kg]	Group 3 (n = 42) [0.10 mg/kg]	Group 4 (n = 43) [0.08 mg/kg]	*p* *
Lower	5.1 (2)	7.0 (3)	9.5 (4)	20.9 (9)	0.085
Within	17.9 (7)	25.6 (11)	42.9 (18)	41.9 (18)	0.037
Upper (Total)	76.9 (30)	67.4 (29)	47.6 (20)	37.2 (16) ^†^	0.001
Upper (Skipped) **	30.8 (12)	27.9 (12)	14.3 (6)	14.0 (6)	0.124

% (n); * Fisher–Freeman–Halton exact test (4 × 2), two-sided; Monte-Carlo 1,000,000 replicates. ** Upper (Skipped) represents the percentage (and number) of cases within the total population. Bonferroni-adjusted pair-wise comparisons (α_adj_ = 0.008, six contrasts per category). Only adjusted p-values are shown. Upper (total)—significant differences were found between ^†^ group 1 and group 4 (*p* = 0.002), group 1 and group 3 (*p* = 0.068), and group 2 and group 4 (*p* = 0.055). Within-range—group 1 vs. Group 3: *p* = 0.107. All remaining contrasts: *p* > 0.308 (not significant).

**Table 4:** Adverse effects during the first three months after transplantation.

Variable	Group 1 (n = 39) [0.15 mg/kg]	Group 2 (n = 43) [0.12 mg/kg]	Group 3 (n = 42) [0.10 mg/kg]	Group 4 (n = 43) [0.08 mg/kg]	*p* *
Nephrotoxicity	4 (10.3)	8 (18.6)	6 (14.3)	8 (18.6)	ns
Neurotoxicity	0 (0)	0 (0)	0 (0)	0 (0)	ns
CMV infection	5 (12.8)	4 (9.3)	4 (9.5)	3 (7.0)	ns
BK infection	3 (7.7)	4 (9.3)	4 (9.5)	4 (9.3)	ns
Admission for bacterial infection	4 (10.3)	3 (7.0)	4 (9.5)	3 (7.0)	ns
Tacrolimus withdrawal	0 (0)	0 (0)	0 (0)	0 (0)	ns

n (%); ns, not significant; Nephrotoxicity, decrease in serum creatinine less than 0.5 mg/dl 72 h after starting tacrolimus; Neurotoxicity, disabling neurotoxicity; CMV infection, cytomegalovirus infection; BK infection, BK virus infection; Tacrolimus withdrawal, need to interrupt tacrolimus treatment due to toxicity. * The data were analyzed using a chi-square test. Values *p* < 0.05 were considered significant.

## Data Availability

The datasets presented in this article are not readily available because the data are part of an ongoing Ph.D. research project.

## Abbreviations

Area under the curve (AUC); Chronic Kidney Disease Epidemiology Collaboration (CKD-EPI); trough concentration normalized by daily dose (C/D ratio); AUC normalized by daily dose (AUC_0–24h_/D ratio); cytochrome P450 family 3 subfamily A (*CYP3A*).
